# Mr. Simmons, on the Fatal Effects of the Cæsarean Operation

**Published:** 1799-10

**Authors:** W. Simmons

**Affiliations:** Manchester


### *To the Editors of the Medical and Physical Journal.*

GENTLEMEN,

THE maxim inculcated by writers on surgery, that a wound of the uterus is mortal, is confirmed by the uniformly fatal event of the Cæsarean section.

The operation is, notwithstanding, insisted on by some practitioners, in a *speculative* case; this, however, they have failed to describe, and the accoucheur is consequently left to conjecture the right application of their doctrine.

It may be useful to enquire into the existence of this supposed case; and also to fix a principle for the government of our conduct.

To

To do this, it will not be neceſſary to enter into nice calculations of the dimenſions of the pelvis; for a general ſtatement of the queſtion will, I think, ſuffice, and lead to an obvious and appoſite concluſion.

I can conceive, that an inciſion might be made into the right ventricle of the heart, and that a polypus might be extracted from its cavity; that the lips of the wound being brought into contact, union by the firſt intention might take place, and the patient recover.

A wound of this organ has, however, proved invariably fatal; ſo that, ſhould ſuch a project be put in execution, the operator might be deemed guilty of murder. The cruelty of ſuch an experiment would not be leſſened by the *poſſibility* of a recovery, as all rational practice muſt reſt on *moral* evidence. To apply this argument to the Cæſarean ſection: Suppoſe the pelvis of a woman to be ſo diſtorted as to prevent the delivery of her child through its contracted aperture, and that it ſhall be certainly known that the child is alive, and ſtrong; as the mother would die undelivered, and the child might be ſaved, would not theſe circumſtances juſtify the performance of the operation?

This, I conceive, conſtitutes the only caſe in which a reaſoning mind would ever entertain a thought of performing it.

All the experience of this country informs us, that the Cæſarean ſection will prove fatal to the mother; and therefore the whole queſtion turns on this ſingle point, Whether the mother's life ſhall be ſacrificed to ſave her child?

I anticipate that the anſwer will, in general, be in the negative; for, beſides that the intention of employing profeſſional aſſiſtance is to ſave, and not to deſtroy; the legiſlature has not thought fit to enact a ſtatute of indemnity for this particular caſe; and the ſixth commandment ſays

"*Thou ſhalt do no* MURDER."

Both divine and human laws then prohibit the employment of means, which will be deſtructive to the parent, though certainly preſervative of the life of her child; and to perform the operation, even in the above-deſcribed caſe, would be to exerciſe a power in oppoſition to thoſe omnipotent authorities.

The queſtion then is ſtopped *in limine*, and our attention muſt be confined ſolely to the mother; as the conſideration of ſaving her child cannot be entertained, without previouſly determining to deſtroy her.

But this is putting the queſtion more favourably than experience warrants, for the ſigns by which we muſt judge of the ſtate of the child before birth, are are inconclusive of its real condition; and, consequently, should the mother's life be yielded to its intended preservation, disappointment might even precede her melancholy catastrophe, in the extraction of a fœtus already dead.

This view of the subject leads to an obvious deduction, that the Cæsarean section is inadmissible during the parent's life; and hence is derived a rule, at once plain and precise, to direct our conduct on this trying occasion; for, when other means fail to accomplish the delivery, or are deemed inexpedient, we can only deplore the miserable sufferings of the patient, and the insufficiency of art to relieve them; and the disposal of life must be left to HIM who gave it.

MANCHESTER, *Aug.* 21, 1799. W. SIMMONS.

